# Delivery of miRNA-29b Using R9-LK15, a Novel Cell-Penetrating Peptide, Promotes Osteogenic Differentiation of Bone Mesenchymal Stem Cells

**DOI:** 10.1155/2019/3032158

**Published:** 2019-04-11

**Authors:** Qiuling Liu, Zhen Lin, Yi Liu, Jiang Du, Hongsheng Lin, Jing Wang

**Affiliations:** ^1^Department of Orthopedics, The First Affiliated Hospital of Jinan University, Guangzhou, Guangdong Province, China; ^2^Affiliated Stomatology Hospital of Guangzhou Medical University, School of Stomatology, Guangzhou Medical University, Guangzhou, Guangdong Province, China

## Abstract

Delivery of osteogenesis-promoting microRNAs (miRNAs) is a promising approach to enhance bone regeneration. In this study, we generated nanocomplexes comprising the novel cell-penetrating peptide R9-LK15 and miR-29b and investigated their effects on osteogenic differentiation of bone mesenchymal stem cells (BMSCs). R9-LK15/miR-29b nanocomplexes were prepared and characterized. The transfection efficiency, cell viability, and osteogenic differentiation were investigated. The results showed that R9-LK15 maintained the stability of miR-29b in serum for up to 24 h. Moreover, R9-LK15 efficiently delivered miR-29b into BMSCs; the transfection efficiency was ~10-fold higher than that achieved using Lipofectamine 2000. The Cell Counting Kit-8 assay showed that R9-LK15 and R9-LK15/miR-29b nanocomplexes had negligible cytotoxic effects on BMSCs. Delivery of R9-LK15/miR-29b nanocomplexes promoted osteogenic differentiation of BMSCs and extracellular matrix mineralization by upregulating alkaline phosphatase expression and downregulating histone deacetylase-4 expression. In general, we developed a novel miRNA delivery system that has a high transfection efficiency and promotes osteogenic differentiation.

## 1. Introduction

MicroRNAs (miRNAs), which contain 20–30 nucleotides, regulate endogenous gene expression by inhibiting posttranscriptional gene expression or inducing degradation of their targets via binding to the 3′ untranslated regions of messenger RNAs (mRNAs) [1–5]. Accumulated evidence suggests that many miRNAs help to regulate osteogenic differentiation of bone mesenchymal stem cells (BMSCs) [3, 6–9]. MiR-29b, one of the most widely investigated miRNAs, regulates osteogenic differentiation via several mechanisms. On one hand, miR-29b decreases protein synthesis and mRNA expression of collagen at the late stage of osteogenesis. On the other hand, miR-29b regulates the Wnt, ERK, and MAPK signaling pathways by targeting their inhibitors at an early stage of osteogenesis. In addition, miR-29b directly downregulates suppressors of osteogenic differentiation, such as histone deacetylase-4 (HDAC4), transforming growth factor *β*3, CTNNBIP1, and DUSP2, by binding to the 3′ untranslated regions of their mRNAs [10–13]. Hence, miR-29b controls osteogenic differentiation by downregulating antiosteogenesis factors and regulating bone extracellular matrix (ECM) proteins.

Unfortunately, noncomplexed miRNAs are negatively charged and thus cannot traverse the plasma membrane, which hinders their clinical application. Moreover, the transfection efficiency of stem cells, such as BMSCs and human autologous adipose tissue-derived mesenchymal stromal/stem cells, is extremely low [14, 15]. Therefore, a delivery system with a high transfection efficiency and low cytotoxicity is required to deliver miRNAs across the plasma membrane and thus promote osteogenic differentiation.

Cell-penetrating peptides (CPPs) are an alternative delivery system. Stable complexes form via binding of positively charged residues in CPPs to the phosphate backbone of nucleic acids [16, 17]. Moreover, CPPs exhibit negligible cytotoxicity and do not elicit an immune response [18, 19]. CPPs are widely used to deliver many molecules, such as proteins, peptides, DNA, small interfering RNAs, miRNAs, and small drugs [20, 21]. Although CPPs-meditated cellular delivery is promising, CPPs are hardly cell-type specific [22]. TAT-derived peptide (TAT) and a polyarginine peptide (R9) have been mostly used [23]. A novel CPP delivery system was also generated by fusing TAT to the amphipathic peptide LK15. The transfection efficiency of TAT-LK15 is significantly higher than those of TAT and LK15 alone [24, 25]. Interestingly, the transfection efficiency of R9 is 20-fold higher than that of TAT [26]. Therefore, we speculated that a novel CPP comprising R9 and LK15 would improve the transfection efficiency of miRNAs and thereby enhance osteogenic differentiation of BMSCs.

In this study, we designed a new CPP (termed R9-LK15) by fusing R9 to LK15. miR-29b was efficiently delivered into the cytoplasm of BMSCs using R9-LK15. The transfection efficiency of R9-LK15 was significantly higher than that of Lipofectamine 2000 (Lipo). Moreover, delivery of miR-29b using R9-LK15 upregulated alkaline phosphatase (ALP) expression and downregulated HDAC4 expression in BMSCs. Our data demonstrate that R9-LK15 facilitates delivery of miR-29b into the cytoplasm and thereby promotes osteogenic differentiation of BMSCs.

## 2. Materials and Methods

### 2.1. Materials

R9 (RRRRRRRRR) and R9-LK15 (RRRRRRRRRGGGKLLKLLLKLLLKLLK) peptides were synthesized by TOP-peptide Co., Ltd. (Shanghai, People's Republic of China). miR-29b (5′-UAGCACCAUUUGAAAUCAGUGUU-3′) was synthesized by GenePharma Co., Ltd. (Shanghai, People's Republic of China). Fluorescein amidite (FAM)-labeled miR-29b was obtained from GenePharma Co., Ltd. Agarose, DNA markers, and GelStain were purchased from TransGen Biotech (Beijing, People's Republic of China). Rat-derived BMSCs were a gift from Professor Gang Wu (Guangzhou Medical University, Guangzhou, People's Republic of China). *α*-Minimum Essential Medium, fetal bovine serum (FBS, Gibco), Lipofectamine 2000, a LIVE/DEAD™ Viability/Cytotoxicity Kit, an anti-rat Alexa Fluor 555-conjugated secondary antibody, and SYBR Green I were obtained from Thermo Fisher Scientific (Waltham, MA, USA). A Cell Counting Kit-8 (CCK-8) was purchased from Dojindo Molecular Technologies Inc. (Tokyo, Japan). Antibodies against *α*-tubulin and RUNX2 were obtained from Abcam (Cambridge, UK) and antibody against HDAC4 was purchased from Cell Signaling Technology (Danvers, MA, USA), respectively. 4′,6-Diamidino-2-phenylindole (DAPI), a Placental Alkaline Phosphatase Assay Kit, and enhanced chemiluminescence reagents were purchased from Beyotime Biotechnology (Nanjing, People's Republic of China). Alizarin Red S, cetylpyridinium chloride, and polyvinylidene fluoride membrane were obtained from Sigma-Aldrich (St. Louis, MO, USA). An RNeasy Mini Kit and RNase-Free DNase Set were purchased from Qiagen (Hilden, Germany). The protease and phosphatase inhibitor cocktail was purchased from MedChemExpress (Monmouth Junction, NJ, USA). A horseradish peroxidase-conjugated goat anti-rabbit secondary antibody was obtained from ABclonal Technology (Wuhan, People's Republic of China). Real time polymerase chain reaction (RT-PCR) primers targeting glyceraldehyde-3-phosphate dehydrogenase (GAPDH) and HDAC4 were purchased from IGE Biotechnology Ltd. (Guangzhou, People's Republic of China). The primer sequences are listed in Table 1.

### 2.2. Preparation and Characterization of miR-29b/R9-LK15 Nanocomplexes

To generate R9-LK15/miR-29b nanocomplexes, 10 *μ*l of 10 *μ*M miR-29b was mixed with increasing volumes of 10 *μ*M R9-LK15. The molar ratio of R9-LK15 and miR-29b was 1:1–7:1. The total volume was adjusted to 200 *μ*l by adding ultrapure water. Thereafter, the mixture was incubated for 1 h at 4°C to generate R9-LK15/miR-29b nanocomplexes. Binding between R9-LK15 and miR-29b was evaluated by 2.0% gel electrophoresis. The size and zeta potential of R9-LK15/miR-29b nanocomplexes were measured by dynamic light scattering using a Zetasizer Nano instrument (Malvern, Westborough, MA, USA). All measurements were performed at 25°C. The morphology of R9-LK15/miR-29b nanocomplexes was evaluated by transmission electron microscopy (TEM; Hitachi Ltd., Tokyo, Japan). The stability of R9-LK15/miR-29b nanocomplexes in fetal bovine serum was assessed by 2% gel electrophoresis. Noncomplexed miR-29b and R9-LK15/miR-29b nanocomplexes were mixed with serum at a volume ratio of 1:1 and incubated at 37°C for various durations. The integrity of noncomplexed and complexed miR-29b dissociated from the complex using loading buffer containing 2%SDS was evaluated by 2% gel electrophoresis.

### 2.3. Cell Culture

BMSCs were cultured in Minimum Essential Medium Alpha Modification supplemented with 10% FBS at 37°C in an incubator containing 5% CO_2_.

### 2.4. Assessment of Cytotoxicity

The cytotoxic effects on BMSCs were evaluated using the CCK-8 assay. Cells were seeded into a 96-well plate at a density of 1 × 10^4^ cells/well and incubated overnight. miR-29b was mixed with Lipo and incubated for 30 min at room temperature. Alternatively, miR-29b was mixed with R9-LK15 and incubated for 1 h at 4°C. Transfection complexes were added to the cells. After 2, 4, or 6 days, 10 *μ*l of CCK-8 solution was added to each well and cells were incubated at 37°C for 1 h. Absorbance at 450 nm was measured using a microplate reader (Thermo Fisher Scientific).

Cytotoxicity was further assessed by the LIVE/DEAD assay. Cells were seeded into a 96-well plate and treated with miR-29b, Lipo, R9-LK15, Lipo/miR-29b nanocomplexes, and R9-LK15/miR-29b nanocomplexes. After 3 days, cells were stained using a LIVE/DEAD™ Viability/Cytotoxicity Kit. Briefly, cells were incubated with 0.5 ml of staining solution containing 0.5 *μ*l of DAPI (1 mg/ml) and 0.5 *μ*l of ethidium homodimer-1 (2.5 mg/ml) for 30 min at 37°C. Signals were visualized by microscopy (FV10i-W; Olympus, Tokyo, Japan) using excitation wavelengths of 405 and 555 nm. For the calculation of cell death rate, total cells were marked with DAPI (blue fluorescence), and dead cells were marked with ethidium homodimer-1 (red fluorescence); cells were then counted using Image-Pro-Plus 6 software. For each group of one experiment, three images were analyzed randomly captured in a blinded manner in each group. For each image, 1500-2000 cells were analyzed. Values were measured from three independent experiments.

### 2.5. Confocal Microscopy

Cells were treated with miR-29b (50 nM), R9-LK15, Lipo/miR-29b nanocomplexes, and R9-LK15/miR-29b nanocomplexes for 48 h, washed thrice with phosphate-buffered saline (PBS, pH 7.2), and fixed with 4% formaldehyde for 15 min. Thereafter, cells were treated with PBS containing 0.1% Triton X-100 for 10 min and then incubated with a rat anti-*α*-tubulin primary antibody overnight followed by an anti-rat Alexa Fluor 555-conjugated secondary antibody (red fluorescence) to label the microtubule cytoskeleton. Nuclei were stained with DAPI (blue fluorescence). Cells were imaged by confocal laser scanning microscopy (Carl Zeiss, Boston, MA, USA) using excitation wavelengths of 405, 488, and 555 nm. Images were acquired at a resolution of 1024 × 1024 pixels and 16-bit depth.

### 2.6. Flow Cytometry

The transfection efficiency of FAM-labeled miR-29b (50 nM) complexed with Lipo and R9-LK15 was quantitated by flow cytometry. Cells were transfected, washed with PBS for 10 min to remove surface-bound fluorescent particles, and lysed. The fluorescence intensity was assessed by flow cytometry (BD FACSVerse, BD Biosciences, Franklin Lakes, NJ, USA). A total of 10,000 cells were analyzed. Data were visualized in logarithmic mode. Untreated cells and those treated with noncomplexed FAM-labeled miR-29b were used as controls.

### 2.7. Assessment of ALP Activity and ECM Mineralization

Cells were grown in 48-well plates and treated with 50 nM noncomplexed miR-29b or miR-29b complexed with Lipo or R9-LK15. Untreated cells and those treated with Lipo and R9-LK15 were used as controls. After 6 h, the medium was replaced by mineralization medium containing 10% FBS, 50 mg/ml ascorbic acid, 10 mM *β*-glycerophosphate, and 0.1 mM dexamethasone. Cells were cultured for another 7 days, washed twice with PBS, fixed with 4% paraformaldehyde, and incubated with ALP working solution at room temperature for 30 min. ALP activity was measured using a commercially available kit according to the manufacturer's instructions.

Alternatively, cells were cultured for another 14 days, incubated in 2% Alizarin Red S prepared in water for 5 min at room temperature, and washed with PBS. ECM mineralization was quantified by extracting Alizarin Red S with 10% cetylpyridinium chloride at room temperature for 2 h and then measuring its absorbance at 570 nm.

### 2.8. Quantitative RT-PCR

Cells were incubated with 50 nM noncomplexed miR-29b or miR-29b complexed with Lipo or R9-LK15 for 48 h. After 7 days, total RNA was extracted using an RNeasy Mini Kit and purified using an RNase-Free DNase Set according to the manufacturer's instructions. Quantitative RT-PCR was performed on an ABI PRISM 7500 system (Applied Biosystems, CA, USA) using SYBR Green I according to the manufacturer's instructions. Primers targeting HDAC4, ALP, and GAPDH were shown in Table 1.

### 2.9. Western Blotting

Cells were treated with 50 nM noncomplexed miR-29b or miR-29b complexed with Lipo or R9-LK15 for 48 h, washed with PBS, and lysed in lysis buffer (25 mM HEPES, 150 mM NaCl, 5 mM EDTA, pH 7.4, 10% glycerol, and 1% Triton X-100) containing a protease and phosphatase inhibitor cocktail. The lysate was resolved by 10% SDS-PAGE, and proteins were transferred to a polyvinylidene fluoride membrane. Membranes were blocked in Tris-buffered saline containing 5% milk and 0.05% Tween and then incubated with primary antibodies at 4°C overnight. After washing, membranes were incubated with a horseradish peroxidase-conjugated goat anti-rabbit or anti-mouse secondary antibody. Signals were visualized using enhanced chemiluminescence reagents.

### 2.10. Statistical Analysis

Data are presented as the mean ± standard deviation (SD). Statistical analyses were performed using a one-way analysis of variance.* P* < 0.05 was considered statistically significant. 

## 3. Results

### 3.1. Characterization of R9-LK15/miR-29b Nanocomplexes

**Figure 1:**
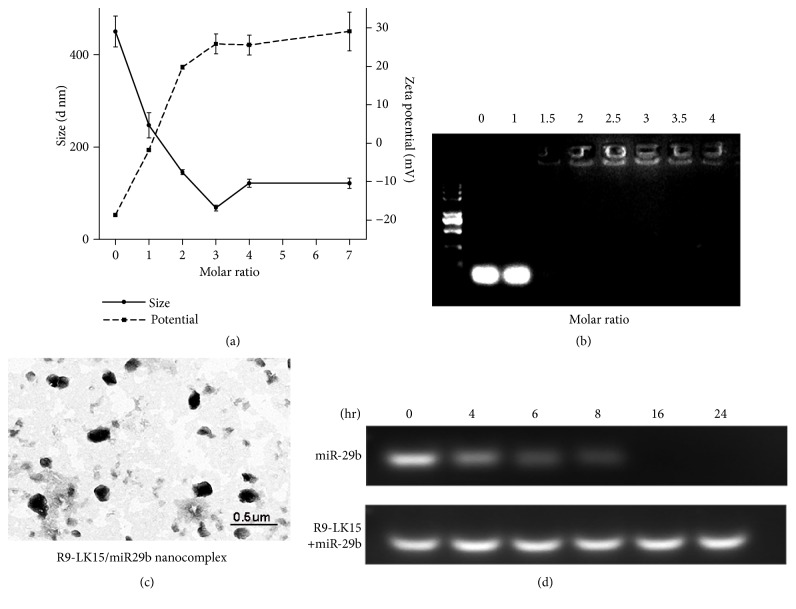
*Characteristics of R9-LK15/miR-29b nanocomplexes.* (a) Dynamic light scattering analysis of the size and zeta potential of R9-LK15/miR-29b nanocomplexes formed by mixing R9-LK15 and miR-29b at various molar ratios. The concentration of miR-29b was 50 nM. (b) A gel retardation assay to determine the formation of nanocomplexes following mixing of R9-LK15 and miR-29b at molar ratios of 1:1–4:1. (c) A TEM image of R9-LK15/miR-29b nanocomplexes. (d) Stability of miR-29b alone and complexed with R9-LK15 upon incubation in serum at 37°C for various durations.

The diameter of R9-LK15/miR-29b nanocomplexes tended to decrease as the molar ratio increased and was lowest (68.13 ± 8.74 nm) at a molar ratio of 3:1 (Figure 1(a), Table 2). The particles might be well distributed due to charge-charge interactions at a molar ratio at 3:1 and then slightly increased with higher molar ratio of R9-LK15. An explanation for this trend is that electrostatic interactions between R9-LK15 and miR-29b result in the formation of R9-LK15/miR-29b nanoclusters from well-distributed colloids. A gel retardation assay was performed to determine whether condensed R9-LK15/miR-29b nanocomplexes formed at a molar ratio of 1:1–4:1. The intensity of the band corresponding to miR-29 decreased as the molar ratio increased, implying that condensed R9-LK15/miR-29b nanocomplexes formed (Figure 1(b)). Consistently, TEM demonstrated that R9-LK15/miR-29b nanocomplexes were condensed (Figure 1(c)). The zeta potential and size of R9-LK15/miR-29b nanocomplexes were optimal at a molar ratio of 3:1, and this ratio was therefore used in subsequent experiments.

We examined the stability of miR-29b in nanocomplexes upon incubation in serum (Figure 1(d)). The intensity of the band corresponding to noncomplexed miR-29b gradually decreased over the first 8 h and disappeared after 16 h. By contrast, the band corresponding to miR-29b complexed with R9-LK15 remained detectable at all time points. These results indicate that R9-LK15/miR-29b nanocomplexes are stable and protect miR-29b against degradation in serum for up to 24 h.

### 3.2. Transfection Efficiency of R9-LK15/miR-29b Nanocomplexes

**Figure 2:**
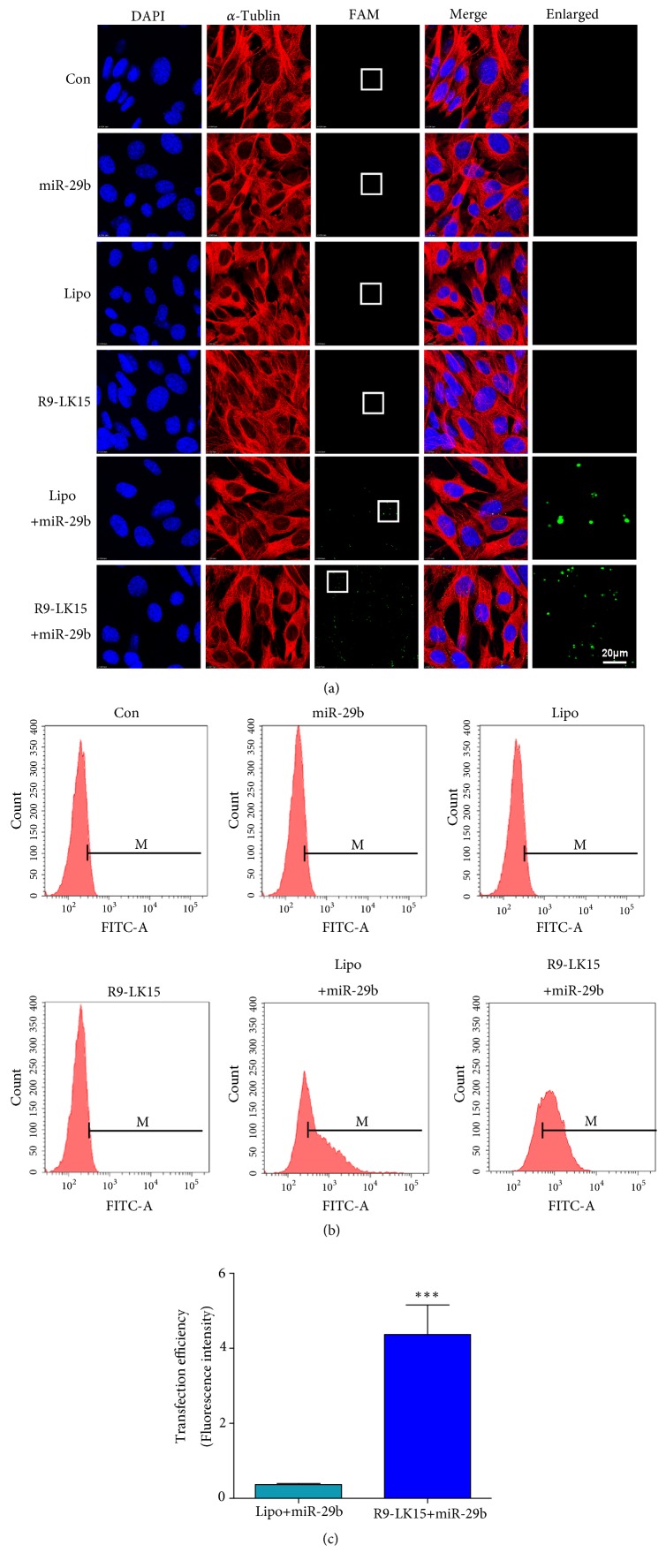
*Transfection efficiency of R9-LK15/miR-29b nanocomplexes.* (a) Confocal microscopy images of BMSCs treated with FAM-labeled miR-29b (50 nM) alone or complexed with Lipo or R9-LK15. Untreated cells (Con) and those treated with Lipo and R9-LK15 served as controls. Scale bar: 20 *μ*m. (b) Flow cytometric analysis of uptake of FAM-labeled miR-29b by BMSCs. (c) Fluorescence intensity of FAM in confocal microscopy images. Data are the mean ± SD of triplicate experiments. ^*∗∗∗*^P < 0.001.

MiR-29b was labeled with FAM to facilitate evaluation of its cellular delivery and complexed with R9-LK15 or Lipo. The final concentration of miR-29b in both complexes was 50 nM. Noncomplexed miR-29b exhibited no intracellular fluorescence, indicating that miR-29b cannot enter cells by itself. However, green fluorescence was observed in cells treated with R9-LK15/miR-29b and Lipo/miR-29b nanocomplexes. Moreover, the number of cells displaying fluorescence was higher upon treatment with R9-LK15/miR-29b nanocomplexes than upon treatment with Lipo/miR-29b nanocomplexes (Figure 2(a)). We evaluated the transfection efficiency by performing fluorescence-activated cell sorting. The fluorescence intensity of FAM was similar in control cells and those treated with miR-29b, Lipo, and R9-LK15 (P>0.05), but was higher in cells treated with R9-LK15/miR-29b and Lipo/miR-29b nanocomplexes. Furthermore, the percentage of positive cells was much higher in samples treated with R9-LK15/miR-29b nanocomplexes (78.33% ± 5.90) than in samples treated with Lipo/miR-29b nanocomplexes (36.43% ± 1.75). Moreover, the fluorescence intensity of FAM was distributed uniformly in cells treated with R9-LK15/miR-29b nanocomplexes than in cells treated with Lipo/miR-29b nanocomplexes, consistent with the confocal microscopy analysis (Figure 2(b)). In addition, the fluorescence intensity of FAM was ~10-fold higher in cells treated with R9-LK15/miR-29b nanocomplexes than in cells treated with Lipo/miR-29b nanocomplexes (P < 0.05, Figure 2(c)).

### 3.3. Effect of R9-LK15/miR-29b Nanocomplexes on Cell Viability

**Figure 3:**
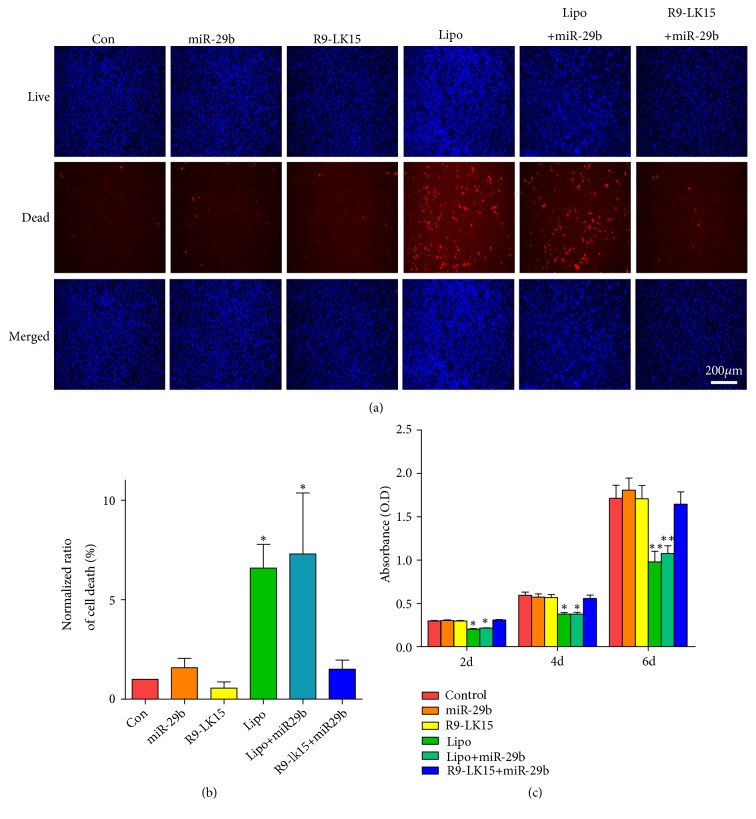
*Cytotoxic effects of R9-LK15/miR-29b nanocomplexes on BMSCs*. (a) LIVE/DEAD assay of untreated BMSCs (Con) and those treated with miR-29b, Lipo, R9-LK15, Lipo/miR-29b nanocomplexes, and R9-LK15/miR-29b nanocomplexes for 72 h. (b) The percentage of dead cells was calculated. Data are the mean ± SD of triplicate experiments. ^*∗*^P < 0.05. (c) CCK-8 assay of untreated BMSCs (Control) and those treated with miR-29b, Lipo, R9-LK15, Lipo/miR-29b nanocomplexes, and R9-LK15/miR-29b nanocomplexes for 2, 4, and 6 days. Data are the mean ± SD of triplicate experiments. ^*∗*^P < 0.05, ^*∗∗*^P < 0.01.

We evaluated cytotoxicity using the LIVE-DEAD assay. Almost no red fluorescence, indicative of cell death, was observed in control cells and those treated with miR-29b, R9-LK15, and R9-LK15/miR-29b nanocomplexes (Figure 3(a)). However, many dead cells were detected in samples treated with Lipo and Lipo/miR-29b nanocomplexes (Figure 3(b)), indicating that these reagents are severely cytotoxic. We evaluated cell proliferation using the CCK-8 assay (Figure 3(c)). After 2, 4, and 6 days, absorbance was significantly lower in cells treated with Lipo and Lipo/miR-29b nanocomplexes than in those treated with R9-LK15/miR-29b nanocomplexes and the other groups (P < 0.05), indicating that R9-LK15 is much less cytotoxic than Lipo.

### 3.4. Effects of R9-LK15/miR-29b Nanocomplexes on Expression and Activity of ALP and ECM Mineralization

**Figure 4:**
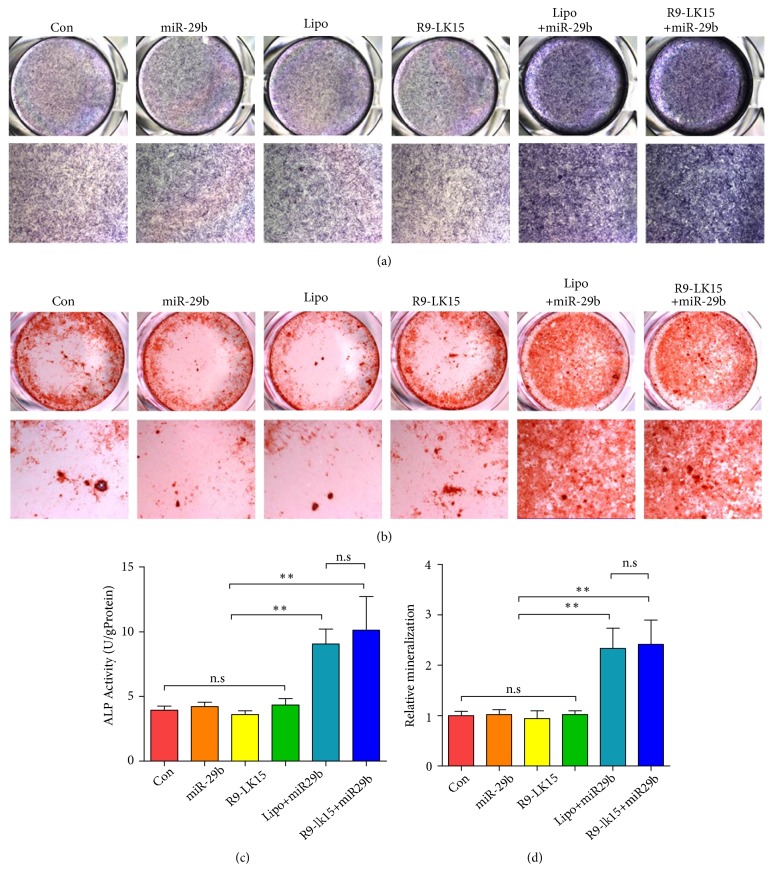
*Effects of R9-LK15/miR-29b nanocomplexes on ALP activity and ECM mineralization.* (a) ALP activity in untreated BMSCs (Con) and those treated with miR-29b, Lipo, R9-LK15, Lipo/miR-29b nanocomplexes, and R9-LK15/miR-29b nanocomplexes for 7 days. (b) Alizarin Red S staining in untreated BMSCs (Con) and those treated with miR-29b, Lipo, R9-LK15, Lipo/miR-29b nanocomplexes, and R9-LK15/miR-29b nanocomplexes for 14 days. (c) Quantification of ALP activity. (d) Quantification of Alizarin Red S staining. Scale bar: 500 *μ*m. ^*∗∗*^P < 0.01.

Osteogenic differentiation of BMSCs was evaluated by measuring expression and activity of ALP and ECM mineralization. ALP activity was similar in control cells and those treated with miR-29b, Lipo, and R9-LK15, but was significantly higher in cells treated with Lipo/miR-29b and R9-LK15/miR-29b nanocomplexes (P < 0.05; Figures 4(a) and 4(c)). Similarly, Alizarin Red S staining, which is indicative of mineralized nodule formation, was similar in control cells and those treated with miR-29b, Lipo, and R9-LK15, but was significantly higher in cells treated with Lipo/miR-29b and R9-LK15/miR-29b nanocomplexes for 14 days (P < 0.05; Figures 4(b) and 4(d)). However, there was no significance between Lipo/miR-29b and R9-LK15 both on activity of ALP and ECM mineralization. These results indicate that transfection of R9-LK15/miR-29b nanocomplexes markedly enhance osteogenic differentiation of BMSCs and ECM mineralization.

### 3.5. Effect of R9-LK15/miR-29b Nanocomplexes on HDAC4 Expression

**Figure 5:**
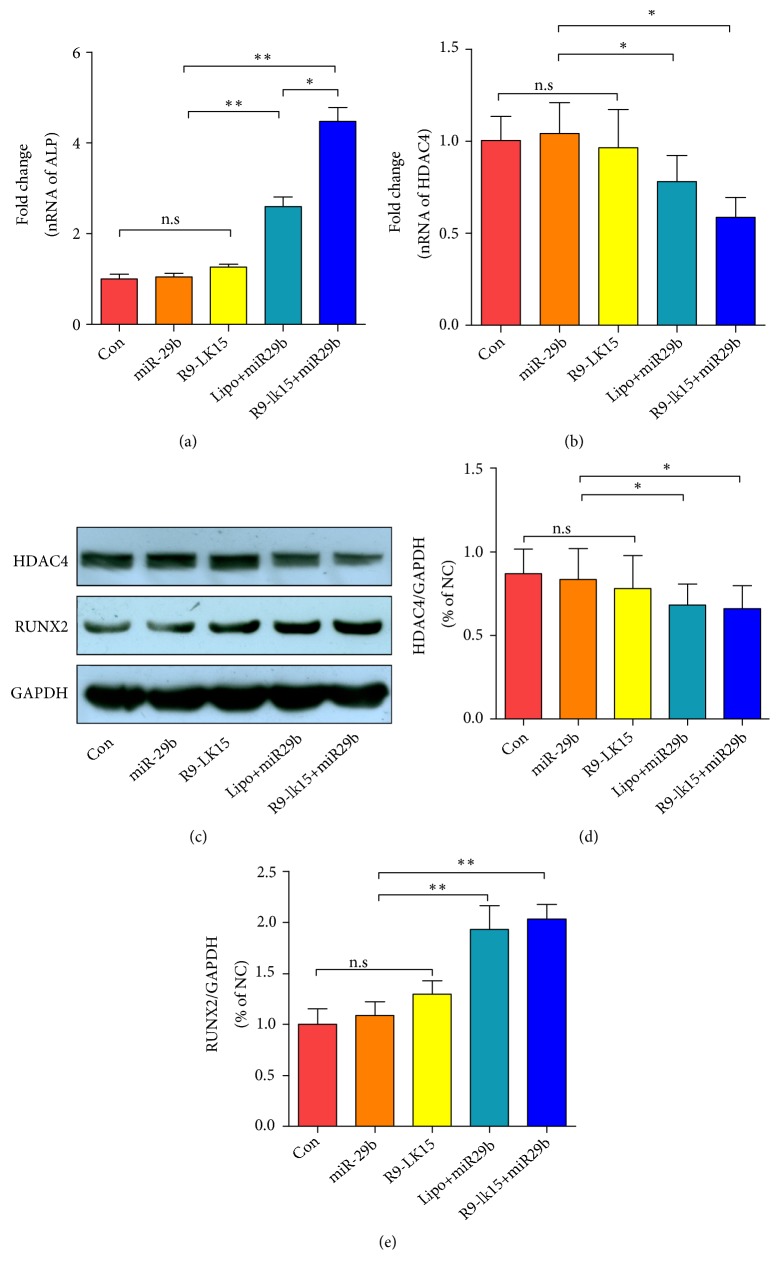
*Effects of R9-LK15/miR-29b nanocomplexes on expression of osteogenesis-related genes and proteins*. (a) RT-PCR analysis of ALP mRNA expression in untreated BMSCs (Con) and those treated with miR-29b, R9-LK15, Lipo/miR-29b nanocomplexes, and R9-LK15/miR-29b nanocomplexes for 7 days. (b) RT-PCR analysis of HDAC4 mRNA expression in untreated BMSCs (Con) and those treated with miR-29b, R9-LK15, Lipo/miR-29b nanocomplexes, and R9-LK15/miR-29b nanocomplexes for 7 days. (c) Western blot analysis of HDAC4 protein expression in untreated BMSCs (Con) and those treated with miR-29b, R9-LK15, Lipo/miR-29b nanocomplexes, and R9-LK15/miR-29b nanocomplexes. (d) Quantification of relative HDAC4 protein expression using ImageJ. Data are the mean ± SD of triplicate experiments. ^*∗*^P < 0.05, ^*∗∗*^P < 0.01.

HDAC4 is the main target of miR-29b and inhibits osteogenesis directly and also indirectly by blocking the p38 MAPK signaling pathway [10, 11]. Firstly, mRNA expression of ALP was significantly higher in cells treated with R9-LK15/miR-29b nanocomplexes than in cells treated with Lipo/miR-29b nanocomplexes (P < 0.05, Figure 5(a)). Moreover, HDAC4 directly interacts with and decreases expression of Runt-related transcription factor 2 (RUNX2) [27]. RUNX2 is vital for bone development that determines the osteogenic phenotype and controls the expression of osteogenic genes such as OPN (Osteopontin), BSP (Bone sialoprotein), and (OCN Osteocalcin) [28, 29]. We evaluated mRNA expression of HDAC4 by performing RT-PCR (Figure 5(b)). mRNA expression of HDAC4 did not markedly differ between control cells and those treated with miR-29b and R9-LK15 (P > 0.05) but was significantly lower in cells treated with Lipo/miR-29b and R9-LK15/miR-29b nanocomplexes (P < 0.05). We further evaluated protein expression of HDAC4 by Western blotting after treatment for 48 h (Figure 5(c)). Consistent with the RT-PCR results, protein expression of HDAC4 was lower in cells treated with Lipo/miR-29b and R9-LK15/miR-29b nanocomplexes than in the other groups (Figure 5(d), P < 0.05). We also evaluated protein expression of RUNX2 by western blotting. Protein expression of RUNX2 was higher in cells treated with Lipo/miR-29b and R9-LK15/miR-29b consistent with HDAC4 expression. These results provide evidence of a promotory effect of R9-LK15/miR-29b on enhancing osteogenesis differentiation of BMSCs.

## 4. Discussion

**Figure 6:**
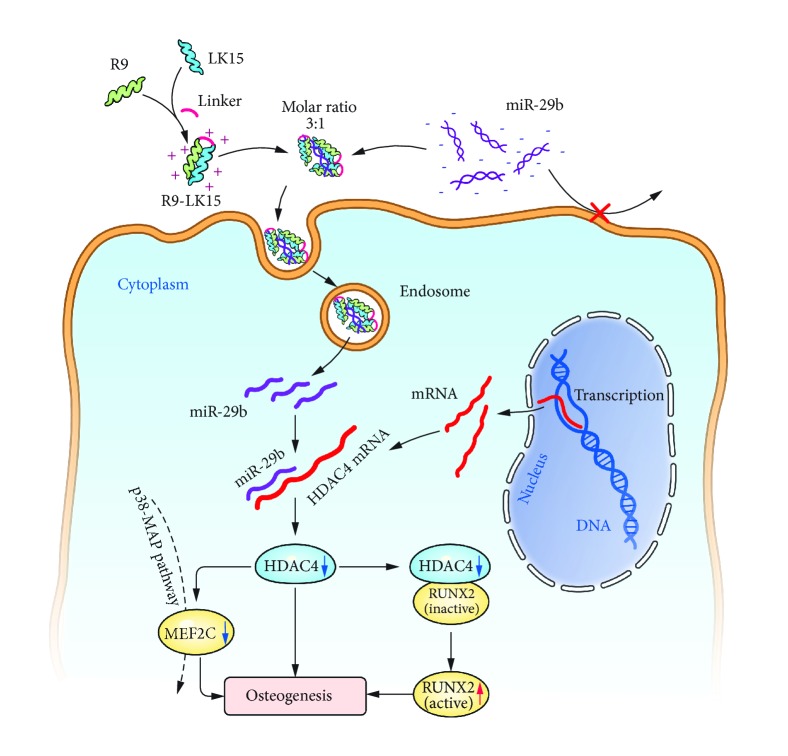
Schematic illustration to explain how transfection of R9-LK15/miR-29b nanocomplexes promotes osteogenesis.

There is increasing evidence that miRNAs are key endogenous regulators of stem cell differentiation [3, 6–9]. Use of miRNA mimics is an excellent strategy to enhance osteogenesis. However, the clinical application of these reagents is hindered by their poor stability and low transfection efficiency [30]. Therefore, a system that efficiently delivers miRNAs while preserving their stability must be developed. In this study, we designed a novel CPP (termed R9-LK15) by linking R9 and LK15 using a triple glycine (GGG) bridge [31, 32]. Nanocomplexes formed by mixing R9-LK15 and miR-29b at a molar ratio of 3:1 had a higher transfection efficiency and were less cytotoxic than Lipo/miR-29b nanocomplexes. Furthermore, R9-LK15/miR-29b nanocomplexes enhanced osteogenic differentiation of BMSCs by downregulating HDAC4 expression. Our results indicate that R9-LK15 is a promising miRNA delivery system and can be used to enhance osteogenic differentiation of BMSCs. The schematic illustration to explain how transfection of R9-LK15/miR-29b nanocomplexes promotes osteogenesis is shown in Figure 6.

The size and zeta potential of delivery systems influence their transfection efficiency [33, 34]. Delivery systems smaller than 200 nm with a zeta potential higher than 20 mV exhibit good stability and high cellular uptake [35]. Nanocomplexes formed by mixing R9-LK15 and miR-29b at a molar ratio of 3:1 had a diameter of 68.13 ± 8.74 nm and a zeta potential of 25.80 ± 4.51 mV. Consistently, TEM indicated that R9-LK15 encapsulated miR-29b to form a stable spherical structure. In addition, R9-LK15 protected miR-29b against degradation in serum for up to 24 h. The enhanced stability of miR-29b is likely due to its tight complexation with R9-LK15.

Lipo is the most commonly used reagent to deliver nucleic acids; however, it is cytotoxic due to its highly positive charge, which restricts its clinical application [36, 37]. The transfection efficiency of R9-LK15/miR-29b nanocomplexes was ~10-fold higher than that of Lipo/miR-29b nanocomplexes. In addition, the CCK-8 assay demonstrated that treatment with Lipo and Lipo/miR-29b nanocomplexes significantly decreased the viability of BMSCs. However, cell viability did not significantly differ between control cells and those treated with R9-LK15 and R9-LK15/miR-29b nanocomplexes. These results indicate that R9-LK15 has a higher transfection efficiency and is less cytotoxic than Lipo. R9-LK15 readily traverses the plasma membrane and is cytocompatible. Together, our results demonstrate that R9-LK15 has marked advantages over Lipo as a gene delivery vehicle.

The effects of R9-LK15/miR-29b and Lipo/miR-29b nanocomplexes on osteogenic differentiation were evaluated. ALP is an early marker of osteogenesis and mediates osteogenic differentiation by promoting ECM mineralization. Calcium deposition occurs after mineralization and is thus a late marker of osteogenesis [38–40]. ALP activity and ECM mineralization were higher in cells treated with Lipo/miR-29b and R9-LK15/miR-29b nanocomplexes than in control cells and those treated with miR-29b, Lipo, and R9-LK15. Moreover, ALP activity and ECM mineralization were higher in cells treated with R9-LK15/miR-29b nanocomplexes than in cells treated with Lipo/miR-29b nanocomplexes. Additionally, mRNA expression of ALP was significantly higher in cells treated with Lipo/miR-29b and R9-LK15/miR-29b nanocomplexes than in the other groups. Induction of osteogenic differentiation upon treatment with Lipo/miR-29b and R9-LK15 nanocomplexes is likely due to their high transfection efficiencies.

We also investigated the mechanism by which R9-LK15/miR-29b nanocomplexes enhance osteogenic differentiation of BMSCs. HDAC4 inhibits osteogenesis by negatively regulating the p38 MAPK signaling pathway and downregulating RUNX2 [11, 13, 41]. mRNA and protein expression of HDAC4 was significantly decreased in cells treated with Lipo/miR-29b and R9-LK15/miR-29b nanocomplexes. In addition, protein expression of RUNX2 was increased in cells treated with Lipo/miR-29b and R9-LK15/miR-29b nanocomplexes. These results indicate that R9-LK15/miR-29b nanocomplexes promote osteogenic differentiation by delivering miR-29b, which suppresses the inhibitory effects of HDAC4 on osteogenesis.

In conclusion, miR-29b is efficiently delivered into the cytoplasm of BMSCs by R9-LK15 and subsequently promotes osteogenic differentiation by downregulating HDAC4 expression. R9-LK15 can be used as a delivery system in gene therapy to enhance intercellular delivery of miR-29b and thereby promote osteogenic differentiation of stem cells. Further studies are required to investigate the effects of this strategy on osteogenic differentiation in vivo.

## 5. Conclusion

We fabricated a novel CPP (termed R9-LK15) to deliver miR-29b into the cytoplasm of BMSCs and thereby promote osteogenic differentiation. R9-LK15 has a higher transfection efficiency and is less cytotoxic than Lipo, suggesting that it has marked advantages over Lipo as a gene delivery vehicle. In summary, this study presents a highly efficient peptide-based miRNA delivery system to promote osteogenic differentiation of BMSCs.

## Figures and Tables

**Table 1:** Target gene primers sequence for real time quantitative PCR.

Gene	Sequence 5'-3'	
GAPDH	Forward: CCTCGTCTCATAGACAAGATGGT	Reverse: GGGTAGAGTCATACTGGAACATG
ALP	Forward: GTGAGCGACACGGACAAGA	Reverse: CCTGGTAGTTGTTGTGAGCATA
HDAC4	Forward: CTGGTATGGGAAGACACAGC	Reverse: AGTCATCTTTGGCGTCGTAC

**Table 2:** The particle size and zeta potential of the miR-29b and R9-LK15 complex.

R9-LK15/miR-29b molar ratio	Particle size (nm)	Zeta potential (mV)
0:1	450.32±57.39	-18.67±0.60
1:1	247.20±47.26	-1.85±0.26
2:1	145.27±9.81	19.77±0.93
3:1	68.13±8.74	25.80±0.93
4:1	121.47±15.12	25.53±4.50
7:1	121.60±19.15	29.07±8.62

## Data Availability

The data used to support the findings of this study are available from the corresponding author upon request.

## Abbreviations

ALP: Alkaline phosphatase
BMSC: Bone mesenchymal stem cell
CCK-8: Cell Counting Kit-8
CPP: Cell-penetrating peptide
DAPI: 4′,6-diamidino-2-phenylindole
ECM: Extracellular matrix
FAM: Fluorescein amidite
FBS: Fetal bovine serum
GAPDH: Glyceraldehyde-3-phosphate dehydrogenase
HDAC4: Histone deacetylase-4
Lipo: Lipofectamine 2000
miRNA: MicroRNA
mRNA: Messenger RNA
PBS: Phosphate-buffered saline
RT-PCR: Reverse transcription polymerase chain reaction
RUNX2: Runt-related transcription factor 2
SD: Standard deviation
TEM: Transmission electron microscopy.
